# Wide temporal horns are associated with cognitive dysfunction, as well as impaired gait and incontinence

**DOI:** 10.1038/s41598-020-75381-2

**Published:** 2020-10-23

**Authors:** Otto Lilja-Lund, Karin Kockum, Per Hellström, Lars Söderström, Lars Nyberg, Katarina Laurell

**Affiliations:** 1 Department of Clinical Science, Neuroscience, Umeå University, Östersunds sjukhus, 831 83 Östersund, Sweden; 2 Institute of Neuroscience and Physiology, University of Gothenburg, Gothenburg, Sweden; 3 Unit of Research, Education and Development, Östersund Hospital, Region Jämtland Härjedalen, Östersund, Sweden; 4 Department of Radiation Sciences, Umeå University, Umeå, Sweden; 5 Department of Neuroscience, Uppsala University, Uppsala, Sweden

**Keywords:** Neurology, Neuroscience, Cognitive ageing

## Abstract

The association between morphology of the brain and symptoms of suspected idiopathic normal pressure hydrocephalus (iNPH) is largely unknown. We investigated how ventricular expansion (width of the temporal horns [TH], callosal angle [CA], and Evans’ index [EI]) related to symptom severity in suspected iNPH. Participants (n = 168; 74.9 years ± SD 6.7; 55% females) from the general population underwent neurological examination, computed tomography, and neuropsychological testing. Multiple linear regression analysis revealed that wide TH was independently associated with all examined iNPH symptoms (*p* < 0.01) except for fine-motor performance, whereas a narrow CA only was associated to specific motor and cognitive functions (*p* < 0.05). TH and EI correlated significantly with incontinence (r_s_ 0.17 and r_s_ 0.16; *p* < 0.05). In conclusion, wide TH was significantly associated with most iNPH-symptoms. This finding potentially reflects the complex nature of the hippocampus, however further studies are needed to demonstrate functional connectivity.

## Introduction

### Clinical manifestations of iNPH, treatment, and prevalence

Idiopathic normal pressure hydrocephalus (iNPH) is a syndrome with a debut typically in the 6th and 7th decade ^1,2^ characterized by balance disturbance and “magnetic shuffling gait” (i.e. decreased step-length and step-height), urinary urgency and cognitive deficiencies such as reduced memory encoding and retrieval, and poor executive function^2–4^. The cause of the disorder is hypothesized to stem from altered cerebrospinal fluid (CSF) resorption^5^ but the underlying pathology is still not fully understood^6^. Even though many questions regarding pathogenesis remain, there is a well-proven neurosurgical treatment with the insertion of a CSF-shunt. Diversion of CSF to the peritoneal cavity or heart is successful in reversing symptoms in up to 80% of the patients^7–9^.

INPH has an estimated prevalence of 3.7% amongst people over 65 years of age^10^. Considering the global trend of prolonged life-expectancy, the societal impact of age-related conditions will have a greater impact in the near future^11^ and efficient treatment is likely to have socioeconomic gains^12^, as well as a good chance of symptom reduction and prolonged longevity^7–9,13,14^. Also, delayed time to surgery may reduce the potential benefits of shunting^15^. It is therefore important for clinicians to detect iNPH at an early stage.

### Neuropsychological and morphological aspects of iNPH

Some of the less known aspects of iNPH is the functional neuropsychology behind the syndrome. The expanding ventricles are exerting mechanical impingement to the corpus callosum, potentially influencing neural function in this area, and contributing to some of the cardinal symptoms of iNPH^16^. The neurocognitive characteristics of psychomotor dysfunction, apathy, and executive symptoms in iNPH are probably related to cortical regions such as the prefrontal cortex and the anterior cingulate cortex (ACC)^2,17–19^. Furthermore, the ACC, the subcortical basal ganglia, and the prefrontal cortex is involved in bladder regulation^20–23^. The caudate nucleus, a part of the basal ganglia, is important in cognition and motor control^22^, and reduced volume of the caudate nucleus and cerebral blood-flow has been associated with iNPH^24,25^. Motor deficiencies other than gait seen in iNPH is reduced fine-motor skills^26^. Memory encoding and retrieval is consistently associated with the hippocampus^27^, and smaller hippocampi are present in iNPH and probably secondary to an increased CSF-volume in the temporal horns (TH)^28^.

Widening of the TH as well as the rest of the ventricular system in iNPH was described by in the first publications of the disorder by Hakim and Adams^29^. The radiological sign of a narrow callosal angle (CA) support that the ventricular dilatation is secondary to hydrocephalus and not to brain atrophy^30^. Another radiological finding of ventricular dilation associated with iNPH is an Evans’ index (EI) above 0.3, which is considered a clinical cut-off when diagnosing iNPH^2^.

This study aimed to describe the relation between the symptoms of iNPH and specific morphological changes. Some cerebral regions hypothesized to contribute to the symptomatology were the ACC^20,21^, the caudate nucleus^22,24^, and the hippocampus^27,28^. These regions were chosen due to their functionality and susceptibility to the morphological changes seen in iNPH^31^. The radiological measures were the width of the TH, EI, and the CA; all of which are recommended when investigating iNPH^2,32^ and have been related to the targeted cerebral regions^30,32–34^.

## Methods

### Subjects

**Figure 1:**
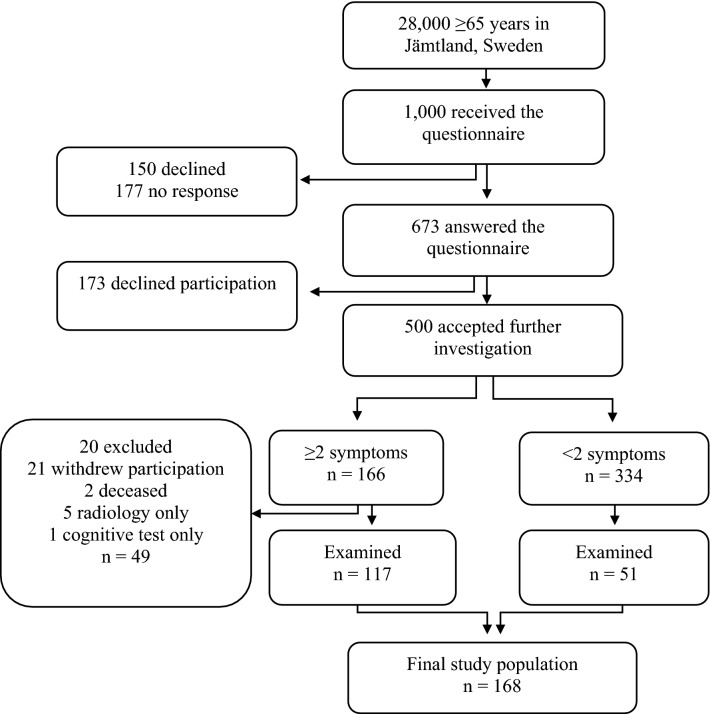
Flow-chart describing selection of participants. The questionnaire consists of seven *yes* or *no* questions regarding presence of iNPH-symptoms.

One thousand randomized individuals over the age of 65 in the county of Jämtland (total population of 28,000), Sweden were invited to participate and to fill out a simple questionnaire with seven questions regarding the presence of typical iNPH symptoms. The questionnaire is presented in Anderson et al.^10^. See Fig. 1 for selection flow-chart. Subjects with known cause for the symptoms, e.g. brain tumor, severe MS, and stroke were excluded. The final sample consisted of 168 individuals with and without symptoms of iNPH. All participants were examined by a senior consultant in neurology. The cognitive testing was performed by the first author who is a specialist in neuropsychology, and a specialist in radiology evaluated the images.

### Measures of symptom-severity

Measures of motor performance included the timed up and go test (TUG)^35^ and the Grooved pegboard test (GPT; Lafayette Instrument Co., Lafayette, IN, USA). On the TUG the participants were asked to get up from a seated position in a chair, walk three meters, turn, walk back, and sit down. The number of steps and time were recorded. On the GPT the participants filled 25 small, grooved holes with a metal pin, each in a unique position, as fast as possible using only one hand^36^.

The Swedish version of the Rey auditory verbal learning test (RAVLT) and Stroop-test^18^ was used in the assessment of verbal memory and executive function respectively. On the RAVLT 15 nouns were read out loud five times. Each time the list of words was read, the participants repeated every remembered word. The total number of correct words was noted. The Stroop-test consisted of two conditions^18^; color-naming and interference. In the color-naming condition, the participant named 100 colored squares (red, green, blue, and yellow) as fast as possible. On the interference-task, the participants read the name of a color, 100 words in total, printed in an incongruent color (e.g., the word ‘red’ is printed in blue) as fast as possible. Incontinence was assessed with a self-rating scale of symptom severity^18^. See Table 1.

**Table 1:** Self-rated symptoms of incontinence.

Rating items		n (%)
1	Normal	64 (38.3)
2	Urgency without incontinence	30 (18)
3	Infrequent incontinence without napkin	44 (26.3)
4	Frequent incontinence with napkin	27 (16.2)
5	Bladder incontinence	0 (0)
6	Bladder and bowel incontinence	2 (1.2)
		167 (100)

Rating items from Hellström et al.^18^.

### Imaging

The participants underwent a non-contrast enhanced computed tomography (CT) of the brain (Philips Ingenuity 2013, 128 channels) within six months of the neuropsychological testing. The protocol used was 120 kV, 400 MaS, rotation time 0.5 s with a pitch of 0.4, generating a slice thickness of 0.6 mm with 4 mm reconstructions in three planes.

**Figure 2:**
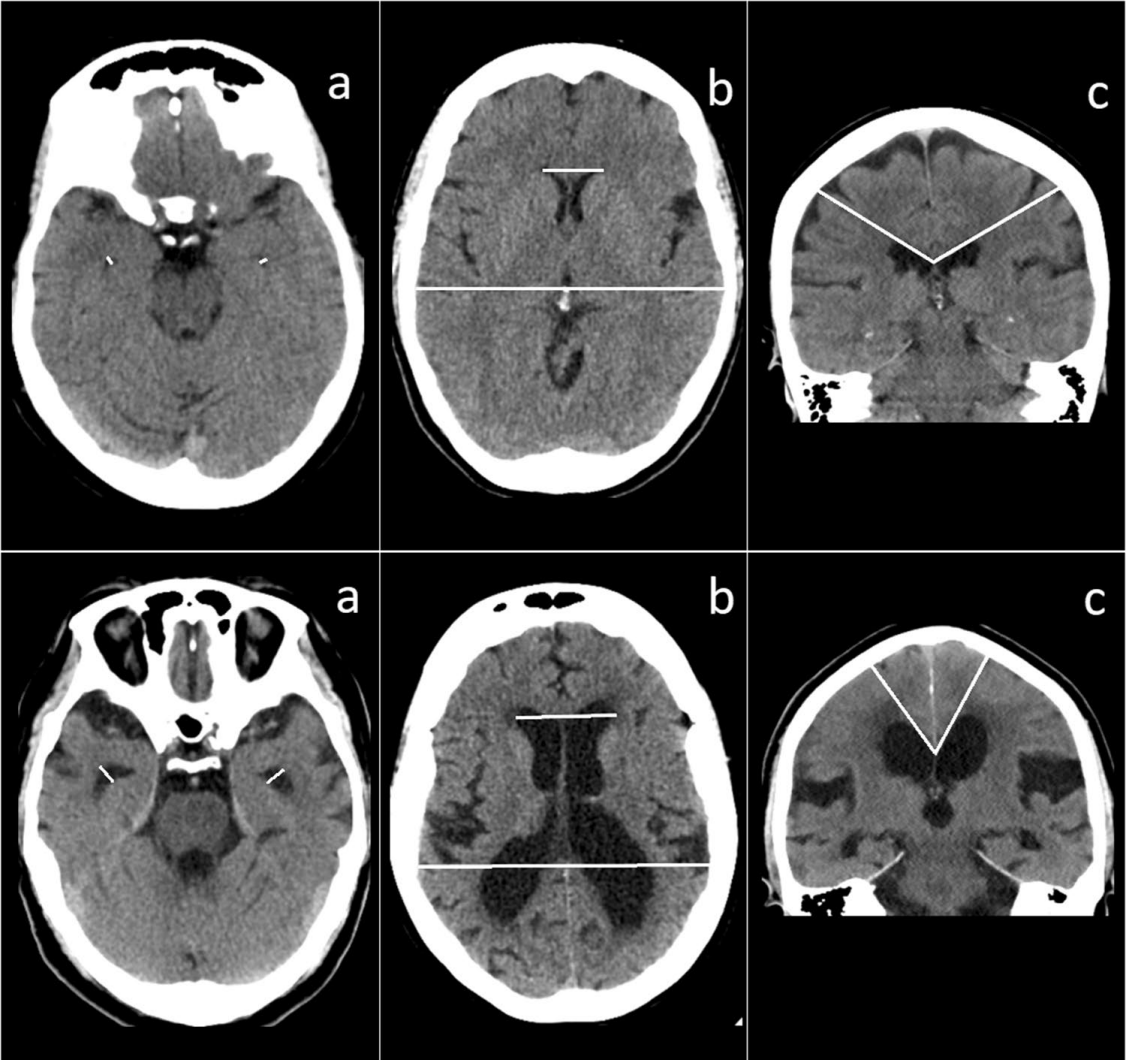
Radiological measurements. The first row shows CT-samples from healthy individuals. The second row represents symptomatic samples. NOTE: Measuring lines in white; (**a**) width of the temporal horns; (**b**) Evans’ index, and (**c**); the callosal angle.

The maximum diameter of the TH was measured in millimeters on each side on transverse images and the average of the left and right TH was calculated^37^. CA was measured between the roofs of the lateral ventricles measured in the coronal plane through the posterior commissure perpendicular to the anterior–posterior commissure plane^30^. EI is the ratio between the maximum frontal horn diameter of the lateral ventricles and the inner diameter of the cranium in the same transverse slice^32^. See Fig. 2 for examples of TH, EI, and CA on CT-images from symptomatic and healthy individuals.

### Statistical analysis

Multiple linear regression analysis using the Enter method were performed on continuous variables. Age and the three radiological markers were predictors in the model, and outcome on the neuropsychological tests were the dependent variables. Tolerance for multicollinearity was controlled and acceptable level was set to > 0.4^38^. Spearman’s rho was used to investigate correlations between the ordinal symptom ratings of incontinence with age and the radiological markers. The level of statistical significance was set to *p* < 0.05. Statistical analyses were conducted using IBM SPSS Statistics 25 (IBM Corp., Armonk, NY, USA).

### Ethical considerations

The study was approved by the Regional Ethical Review Board in Umeå (2014/180-31). All participants gave written informed consent. All methods and procedures were performed in accordance with relevant guidelines and regulations. Clinical follow-up outside the research program was offered to those who showed symptoms in need of further investigation.

## Results

There was an even ratio of males (n = 73) to females (n = 98) in the sample and the age distribution was similar for both genders (males m = 75.3, SD = 6.8; females m = 74.6, SD = 6.5). See Table 2.

**Table 2:** Outcome in the study population.

	n	m	SD
Age in years	168	74.9	6.7
**Computed tomography**			
TH	168	3.6	1.6
EI	168	0.29	0.03
CA	168	113.4	16.3
**Motor performance**			
TUG time	167	12.0	6.2
TUG steps	167	15.9	5.5
GPT best time	132	95.2	35.4
**Cognition**			
RAVLT total score	165	30.3	10.2
Stroop color-naming	163	79.4	25.8
Stroop interference	155	162.6	60.3

*SD* standard deviation; *TH* width of the temporal horns in mm; *EI*  Evans’ index; *CA* callosal angle in degrees; *TUG* timed up and go test in seconds (time) and number of steps; *GPT  * grooved pegboard test in seconds; *RAVLT * Rey auditory verbal learning test with number of words. Stroop results are presented in seconds.

The multiple linear regressions revealed independent relationships between age, symptoms, and radiology (*p* < 0.001), see Table 3. The radiological parameter TH was significantly related to all results of cognition and motor performance (*p* < 0.01) except for GPT. CA was related with results on TUG time and Stroop-interference (*p* < 0.05). No significant relations were found between EI and performance on any of the neuropsychological tests.

**Table 3:** Standardized coefficients and adjusted R^2^ from the multiple linear regression analysis.

Dependent variables (n)	Age			Temporal horns			Evans’ index			Callosal angle			Regression		
	$\upbeta$	SE	*p*	$\upbeta$	SE	*p*	$\upbeta$	SE	*p*	$\upbeta$	SE	*p*	R^2^_adj_	SE	*p*
**Motor performance**															
TUG time (167)	0.22	0.07	0.005	0.28	0.34	0.002	0.03	13.26	0.729	− 0.13	0.03	0.071	0.22	5.47	< 0.001
TUG steps (167)	0.27	0.06	0.001	0.33	0.29	0.001	− 0.10	11.41	0.203	− 0.15	0.02	0.038	0.26	4.71	< 0.001
GPT best time (132)	0.42	0.47	0.001	0.11	2.24	0.263	0.06	83.09	0.527	0.07	0.19	0.355	0.22	31.33	< 0.001
**Cognition**															
RAVLT total score (166)	− 0.19	0.12	0.017	− 0.25	0.56	0.006	− 0.12	21.89	0.128	0.12	0.05	0.110	0.22	8.98	< 0.001
Stroop color-naming (163)	0.05	0.32	0.579	0.28	1.59	0.003	0.01	59.25	0.927	− 0.15	0.12	0.052	0.12	24.14	< 0.001
Stroop Interference (155)	0.17	0.74	0.032	0.25	3.72	0.007	0.09	134.89	0.265	− 0.18	0.28	0.019	0.21	53.50	< 0.001

*SE* standard error; *TUG* timed up and go test; *GPT* grooved pegboard test; *RAVLT* Rey auditory verbal learning test.

Incontinence correlated significantly with age (r_s_ 0.30, *p* < 0.001); TH (r_s_ 0.17, *p* = 0.025); and EI (r_s_ 0.16, *p* = 0.035), but not with CA. See Table 4.

**Table 4:** Spearman’s rho coefficients from incontinence ratings correlated with age and radiological markers.

	Incontinence (n = 167)	
	r_s_	*p*
Age	0.30	< 0.001
TH	0.17	0.025
EI	0.16	0.035
CA	− 0.02	0.769

*TH* temporal horns; *EI* Evans’ index; *CA* callosal angle.

## Discussion

The aim of the study was to investigate the association of ventricular dilation related to the typical symptomatology found in suspected iNPH. We found that poorer performance on tests of cognition, gait, and more symptoms of incontinence were related to increased TH width. These differences remained after controlling for age in the multiple linear regression analysis.

The width of the TH was used as a surrogate for hippocampal anomalies, a structure well known for its involvement in memory function^27^. The results on the RAVLT confirmed that poor memory was related to broader TH. In Alzheimer’s disease (AD), the common finding of wide TH is probably due to atrophy of the hippocampus whereas in hydrocephalus it is more likely that the hippocampus is compressed by the increased CSF volume in the TH. In both cases a reduced function is part of the syndromes.

The hippocampal complex is involved in cognitive functions beyond episodic memory such as spatial mapping and multi-modal processing^27,39^. Navigation and walking are multimodal tasks tapping into spatial orientation and coordination of movement. Studies have found that broader TH were associated to gait instability in healthy elderly^40^ and that reduced gray-matter integrity of the hippocampus, as well as the ACC, was associated with increased step-length variation^41^. The involvement of the hippocampus with increased TH in reduced gait performance coincides with our findings of decreased performance on the TUG-test. In addition, wider TH correlated significantly with symptoms of incontinence. The correlation was weak but raises the question whether cerebral regions in the proximity can be related to micturition control. Urgency incontinence is related to detrusor over-activity and reduced inhibitory regulation during resting state could result in an overactive bladder as manifested in iNPH^42,43^. Other studies have shown that the parahippocampal gyrus is engaged in suppressing the voiding mechanisms at the level of the midbrain periaqueductal grey^42^. Hence, reduced function in the parahippocampal gyrus might be associated with difficulties inhibiting the detrusor muscle, a prominent symptom in patients with iNPH as well as AD.

Decreased volume of the hippocampal formation and increased width of the TH have been related to age in a healthy study population and to mild cognitive impairment (MCI)^44–46^. Future studies on standardized measures of the width of the TH would be helpful to differentiate between pathology and normal ageing. Our study showed that age related to all outcome measures, except for Stroop color-naming test, and age was the variable that had the strongest correlation with symptoms of incontinence as well. Furthermore, age was the only independent variable left with a significant contribution to poor performance on the GPT. Hence, fine-motor skills were not associated to radiological measures of ventricular expansion the way gross-motor skills were.

The EI is used in assessing ventricular expansion and an EI > 0.3 is an obligatory sign when diagnosing iNPH^2,47^. Linking specific symptoms to the EI might be of interest to understand the symptomatology of iNPH. With the caudate nucleus located periventricular to the frontal horns, increased EI was hypothesized to be associated with decreased function engaging the caudate nucleus. However, we could not establish any significant relationships between decreased cognition or motor performance and EI. Nonetheless, there was a significant, albeit weak, correlation with symptoms of incontinence and EI. Although several cerebral regions are engaged in micturition control, including the basal ganglia, this finding needs to be interpreted cautiously in relation to the role of the caudate nucleus.

The CA was related to performance on the Stroop interference task in line with our prediction. This coincide with other findings of ACC regulation on the Stroop-test^48^ and highlight the importance of investigating possible structural changes beyond the pre-frontal cortex when considering executive dysfunctions. However, results on the Stroop color-naming condition did not reveal any significant relationships. This is coherent with the view that the ACC is engaged in conflict monitoring during the interference test^48^, a task that the color-naming condition does not tap into. The number of steps was associated with the CA on the TUG-test. Hence, more steps were needed for the same task. Shortened step-length is one of the characteristics of the “magnetic shuffle” found in iNPH^3,4^. Brodoefel et al.^49^ found reduced gait performance relating to thickness of the corpus callosal genu. Furthermore, corpus callosum impingement was found to differentiate between those with and those without gait impairment in 40 hydrocephalic patients^16^. In summary regarding the CA, we found support for its contribution in some ACC related functions; foremost in conflict monitoring and some gait related deficiencies. However, we did not find that a narrow CA related to incontinence.

Three persons had asymptomatic ventriculomegaly (AVIM)^10^, a possible precursor to iNPH^50^, and are of interest in forthcoming longitudinal studies.

### Limitations

The population-based material in this study is both a strength and a limitation. The risk of selection bias is less compared to a clinical material whereas some of our negative results might be because the majority had few symptoms and normal CT scans.

We had some difficulties procuring the test-board required for the GPT which resulted in some missing data on that specific test. Tests included in this study were chosen on the basis of their relevance in detecting iNPH-symptoms^18^. Other neuropsychological tests could cast light on a wider range of cognitive abilities; but also on the underlying dysfunction causing poorer results on the verbal memory test (such as verbal memory-span and memory consolidation)^36^.

The radiological markers used in our study are related to symptoms of iNPH, but we were neither intending nor able to prove a functional connection. To study such connection functional imaging technique would be more helpful. However, the well-established imaging technique used in this study (CT-brain) is reliable for morphological evaluation^34^, and have clinical relevance due to the availability and comparatively low cost^51^.

## Conclusion

The width of the TH independently related to memory, gross motor- and executive function; and CA to executive dysfunction and gait. Furthermore, wide TH and increased EI correlated with incontinence. A common characteristic for the ventricular deformation investigated in this study is the proximity to cerebral regions engaged in multi-modal processing related to symptoms of iNPH. However, the functional connection needs to be studied further. More normative, and longitudinal studies, targeting pathological and age-related changes of the TH, EI, and CA could potentially clarify early development of iNPH.
